# Scene Recognition for Indoor Localization Using a Multi-Sensor Fusion Approach

**DOI:** 10.3390/s17122847

**Published:** 2017-12-08

**Authors:** Mengyun Liu, Ruizhi Chen, Deren Li, Yujin Chen, Guangyi Guo, Zhipeng Cao, Yuanjin Pan

**Affiliations:** 1 State Key Laboratory of Information Engineering in Surveying, Mapping and Remote Sensing (LIESMARS), Wuhan University, Wuhan 430079, China; amylmy@whu.edu.cn (M.L.); guangyi.guo@whu.edu.cn (G.G.); godsay1983@whu.edu.cn (Z.C.); pan_yuanjin@163.com (Y.P.); 2 Collaborative Innovation Center of Geospatial Technology, Wuhan University, Wuhan 430079, China; 3 School of Geodesy and Geomatics, Wuhan University, Wuhan 430079, China; yujin.chen@whu.edu.cn

**Keywords:** indoor scene recognition, deep learning, indoor localization, WiFi, magnetic field strength, particle filter, smartphone

## Abstract

After decades of research, there is still no solution for indoor localization like the GNSS (Global Navigation Satellite System) solution for outdoor environments. The major reasons for this phenomenon are the complex spatial topology and RF transmission environment. To deal with these problems, an indoor scene constrained method for localization is proposed in this paper, which is inspired by the visual cognition ability of the human brain and the progress in the computer vision field regarding high-level image understanding. Furthermore, a multi-sensor fusion method is implemented on a commercial smartphone including cameras, WiFi and inertial sensors. Compared to former research, the camera on a smartphone is used to “see” which scene the user is in. With this information, a particle filter algorithm constrained by scene information is adopted to determine the final location. For indoor scene recognition, we take advantage of deep learning that has been proven to be highly effective in the computer vision community. For particle filter, both WiFi and magnetic field signals are used to update the weights of particles. Similar to other fingerprinting localization methods, there are two stages in the proposed system, offline training and online localization. In the offline stage, an indoor scene model is trained by Caffe (one of the most popular open source frameworks for deep learning) and a fingerprint database is constructed by user trajectories in different scenes. To reduce the volume requirement of training data for deep learning, a fine-tuned method is adopted for model training. In the online stage, a camera in a smartphone is used to recognize the initial scene. Then a particle filter algorithm is used to fuse the sensor data and determine the final location. To prove the effectiveness of the proposed method, an Android client and a web server are implemented. The Android client is used to collect data and locate a user. The web server is developed for indoor scene model training and communication with an Android client. To evaluate the performance, comparison experiments are conducted and the results demonstrate that a positioning accuracy of 1.32 m at 95% is achievable with the proposed solution. Both positioning accuracy and robustness are enhanced compared to approaches without scene constraint including commercial products such as IndoorAtlas.

## 1. Introduction

After decades of research, there are still no pervasive products for indoor localization while the demand for indoor localization-based service is increasing rapidly in smart cities [1]. Recent years have witnessed a lot of work on indoor localization. Most of them try to provide a widely used scheme for indoor localization and achieve satisfying performance like GPS in outdoor environments. Among these [2,3,4,5,6], fingerprinting-based methods are the most popular due to their effectiveness and independence of infrastructure. Generally, fingerprinting-based methods include Wi-Fi and magnetic fingerprinting. Both of them are based on an assumption that each location has a unique signal feature. The system of fingerprinting localization is usually divided into two phases: offline training and online processing. In the offline phase, Wi-Fi-received signal strength (RSS) or magnetic field strength (MFS) at different reference points are collected to build a radio map. In the online phase, users can sample RSS or MFS data at their positions and search similar signal patterns in the database. The corresponding location with the most similar pattern is considered as a positioning result.

However, although this kind of scheme is easy to be established and can achieve fine performance in the first few months, it still is unable to become a wildly used solution in the location-based service (LBS) market due to these challenges: (a) Data collection is labor-intensive and time-consuming. Surveyors need to collect enough signal samples at every reference point to build the fingerprint database. (b) Database maintenance is difficult, since signal patterns change over time and are vulnerable to environmental changes. In order to guarantee the accuracy of the indoor localization system, fingerprint database updating is required in an appropriate time interval, which is difficult and time-consuming. (c) Ambiguities in positioning results for the reason that different locations may have same signal patterns, especially for magnetic fingerprints. In most of the time, single source of signal is not enough to guarantee the accuracy of positioning result. (d) Low efficiency in fingerprint database matching. When the positioning area is considerable wide, the fingerprint database may be too large to give a positioning result in real-time on commercial smartphones. 

To overcome the drawbacks of traditional fingerprinting-based indoor localization, researchers have proposed a large body of solutions. Among these solutions, a calibration-free mechanism which leverages motion information of users has been formed [4,5,6,7]. Whereas, the accuracy and robustness of these systems are not enough for wide use due to the complexity and diversity of indoor environments. To solve these problems, this paper presents a scheme which fully utilizes built-in sensors on a smartphone, including camera, accelerometer, gyroscope, compass, magnetometer and WiFi. Inertial sensors such as accelerometer, gyroscope and compass are used to record users’ trajectories by pedestrian dead-reckoning (PDR). Every trajectory is construct by a step vector and every step consist of heading, WiFi RSS vector and magnetic field strength (MFS) vector. These steps are used to replace reference points in former fingerprinting-based systems. That is to say, in the proposed system, a fingerprint database can be built by walking trajectories of a user. A surveyor is not required to stay and wait at a reference point to collect signal samples. In this way, difficulties in database construction and maintenance are greatly decreased. Furthermore, inspired by our own spatial cognition experience in indoor environments, the “eye” (camera) of a smartphone is used to “see” the scene around a user. This procedure will give a priori information for localization. With scene information constrained, the searching range of fingerprint database is narrowed down and signal ambiguities are remarkably decreased as a result. To utilize scene information, every trajectory will be attached with a scene label in the database construction phase. To recognize a scene, an indoor scene model will be trained via fine-tuned deep convolutional neural networks (CNNs). The training data is captured by the camera on a smartphone. In the positioning phase, scene recognition will be first conducted. Then, a matching procedure will be carried out to narrow the fingerprint database to a small area (only contain fingerprints in that scene). Finally, a user’s location will be estimated by a particle filter algorithm which fuses WiFi and MFS signals.

The main contributions of this paper can be concluded as follows.

(1) To imitate the visual cognition ability of human, a scene recognition module implemented with deep learning is adopted to improve the accuracy and robustness of infrastructure-free indoor localization system.
(2) A practical indoor localization scheme is designed which digging the full potential of built-in sensors on a smartphone. Particle filter algorithm is used in sensor data fusion.
(3) The database construction in this paper is labor-saving and easily to be extended as a crowdsourcing solution for the reason that data is automatically collected while users’ walking.
(4) The proposed system is implemented on Android platform and performance is evaluated in a challenging indoor environment which includes several glass walls and a patio.

The rest of this paper is organized as follows. Related works are reviewed in Section 2. System architecture and methodology in proposed system are demonstrated in Section 3. Experiments and performance evaluations are presented in Section 4. Section 5 and Section 6 are discussion and conclusion of the whole work and give suggestions in future research.

## 2. Related Work

The literature on fingerprinting-based indoor localization is long and rich. He et al. [8] overviews recent advances on WiFi fingerprinting localization. The survey includes two aspects. One is how to make use of signal patterns, user collaboration and motion sensors. Another is how to reduce labor-intensive database construction, maintain a fingerprint database and calibrate heterogeneous devices. Moreover, Yang et al. [9] supplies a comprehensive literature review on how mobility information enhances smartphone-based indoor localization. These studies indicate that sensor-fusion-based methods are main trends of future pervasive indoor localization services.

Earlier work for sensor-fusion-based indoor localization method can go back to [7], which utilizes commercial smartphones only. Although this system provides a reliable step detection and heading estimation algorithm, it requires users to provide initial location on an indoor map. LiFS systems [5] need little intervention to be deployed since mapping between fingerprint space and stress-free floor plan is created. In this process, mobility information is used to transform a floor plan to a stress-free floor plan and signal patterns are used to extract indoor space features including corridor and room. A more flexible deployment was proposed by Zheng et al. [10], which is a self-deployable system depending on trajectories recorded by guiders. In this system, followers can be navigated to their destination by recorded trajectories. Other similar studies also include [11,12,13,14]. Chen et al. [11] combined WiFi, pedestrian dead-reckoning (PDR) and landmarks with a Kalman filter algorithm. Liu et al. [12] employed spatial correlation extracted from user’s motion. Chen et al. [13] adopted a maximum likelihood-based fusion algorithm to process WiFi and PDR information. While Shu et al. [14] use local disturbances of a geomagnetic field as features and incorporate WiFi signals to achieve a relatively high accuracy. 

In the computer vision community, scene recognition has attracted lots of attention for it is a highly valuable perceptual ability of indoor mobile robots. Previous studies relate to indoor scene recognition including [15,16,17,18,19,20,21,22]. In [22], local and global information are combined to extract indoor scene prototype. In terms of practical training for deep CNNs, references [23,24,25,26,27] give different architectures for different vision tasks. Generally, with a large enough dataset, increasing the capacity of network can improve recognizing accuracy. Krizhevsky et al. [27] designed an eight-layer network to classify 1000 classes in ImageNet with a low error rate. Girshick et al. [23] proposed R-CNN (regions with CNN features) to improve object detection performance and achieved a remarkable enhancement. He et al. [24] allowed CNNs input arbitrary-size images with a spatial pyramid pooling strategy. References [25,26] devoted to understand features in hidden layers. Zhou et al. [25] showed that object detectors emerged inside CNNs trained for scene recognition. Yosinski et al. [26] studied how features in different layers affect the results of transfer learning based on a deep CNN. Considering recognition accuracy and efficiency, an eight-layer architecture is appropriate for our task.

Compared with previous schemes, this paper combines not only WiFi, PDR and geomagnetic features but also cameras to implement a high performance indoor localization system. The camera is adopted to extract the whole semantic information of locating area which noted as indoor scenes. Ref. [28] also combined an optical camera on a smartphone. However, the use of visual information in that work is different from the proposed system. They extracted SIFT features to calculate image matching factors along with orientation sensors. While this paper adopts deep CNNs to recognize indoor scenes without any feature extraction which needs high-cost computation. To evaluate the performance, fingerprint database construction in this paper is similar to a trajectory-based system which is quite popular in both academia [4,5,6,7,10] and industry [29]. The major difference between the proposed system and other trajectory-based systems is that they have not taken visual information into account in localization process. For human experience, visual ability is important for us to locate ourselves and navigate. Applying this useful information to localization systems is helpful to improve the performance. In the proposed system, image data are simultansously collected with other sensors such as WiFi, magnetometer, accelerometer, gyroscope and compass. Trajectories are collected and organized with scene labels. These scene labels can be used to give a boundary of fingerprint database in the localization phase. 

Compared to other indoor scene recognition approaches, scene definitions in this paper are different since the aim of the proposed system is to accurately locate a user. Indoor scenes are labeled combined with localization areas in this paper. While other approaches label them based on their functionalities, such as corridor, bookstore, bedroom, library, gym and so on. In this system, not only category of a scene can be recognized, but also a specific one can be located. Regarding recognition methods of indoor scenes, deep learning is taken into account in this paper. Compered to [22], deep learning is more simplified since it is not necessary to define local or global features. The outputs of hidden layers can be regarded as features which are computed by feedforward neural networks. However, since deep learning is a data-hungry method, transfer learning can be adopted to overcome deficits of training data. In [15], Oquab et al. transferred image representations learned with CNNs on ImageNet dataset to other vision tasks. Besides, Espinace et al. [21] has proven that contextual relations of objects and scenes can facilitate indoor scene recognition. Therefore, a fine-tuned CNN is adopted to train the indoor scene recognition model in this paper, which is based on a pre-trained model with a large-scale dataset for object recognition [30]. In this way, even without a considerable dataset, an indoor scene model can still be trained to recognize a scene with high accuracy.

## 3. System Overview and Methods

In this section, an overview of the system is presented. Then, key modules and important algorithms are described, which include data acquisition, fingerprints processing, scene model training and location estimation.

### 3.1. System Overview

**Figure 1:**
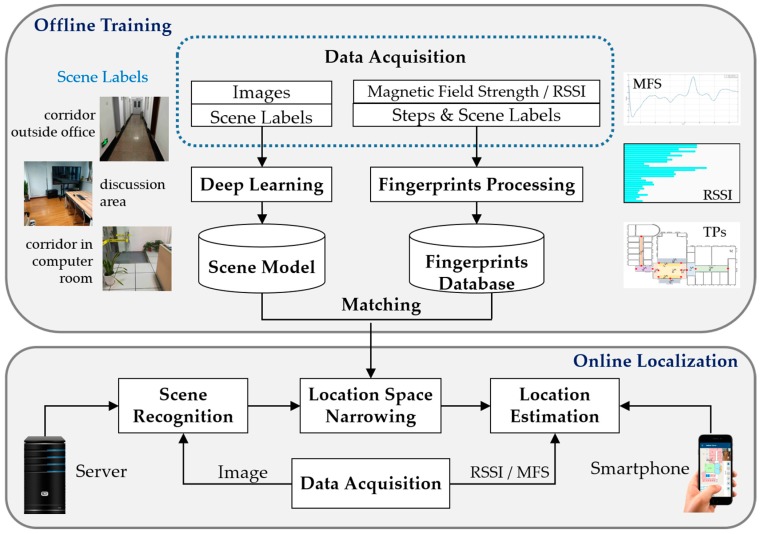
System architecture. MFS, RSSI and TPs refer to convolutional neural networks, magnetic field strength, received signal strength indicator and transition points, respectively.

The architecture of the proposed system is shown as Figure 1. The whole procedure is similar to traditional fingerprinting localization schemes which contain two phases: offline training and online localization. In the offline phase, not only a fingerprint database should be built, but also an indoor scene recognition model will be trained through deep convolutional neural networks. In the online phase, scene recognition will be executed before location estimation to give a constraint for localization. We define scene labels in areas where it is hard to discriminate WiFi signal patterns or magnetic signal patterns. For instance, WiFi fingerprinting often fails to locate a user correctly in an open area such as a patio or a wide hall. While magnetic fingerprinting usually confuses locations for the reason that there are only three dimensions for magnetic measurements from the magnetometer in smartphones. More specifically, in the offline phase, the modules in the proposed system include data acquisition, fingerprint processing and indoor scene model training. In the online phase, the system includes scene recognition, location space narrowing and location estimation.

### 3.2. Data Acquisition and Fingerprints Processing

In this part, details of data acquisition and fingerprint processing are described. Both of these procedures are implemented on a smartphone.

#### 3.2.1. Data Acquisition

**Figure 2:**
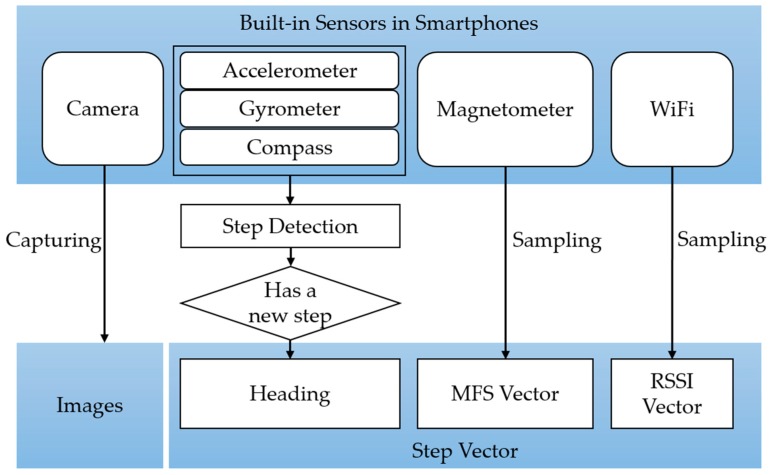
Data acquisition procedure while walking. Note that, WiFi RSSI vector is not required in every step, for its sampling frequency is slower than other sensors. If WiFi is not available, it will be set to null in a step vector.

In the stage of data acquisition, images are automatically captured along with the WiFi received signal strength indicator (RSSI) and magnetic field strength (MFS). In the meantime, a step detection module following [7] will be activated and heading of every step will be recorded. The illustration of data acquisition while walking is shown as Figure 2. 

When a step has been detected in a scene, the position of that point can be computed as:

(1) $$\{\begin{array}{c} x_{i+1}=x_{i}+stepLength*\cos\left(r_{i}\right)\\ y_{i+1}=y_{i}+stepLength*\sin\left(r_{i}\right) \end{array}$$

where $r_{i}$ is the heading of $step_{i}$ and stepLength can be predicted by an empirical model using Equation (2) proposed by [31].

(2) $$stepLength=\left(0.7+a\left(H-1.75\right)+\frac{b*\left(StepFrequency-1.79\right)*H}{1.75}\right)*c$$

a,b and c are model parameters for each person and can be calibrated by pre-training. 

**Figure 3:**
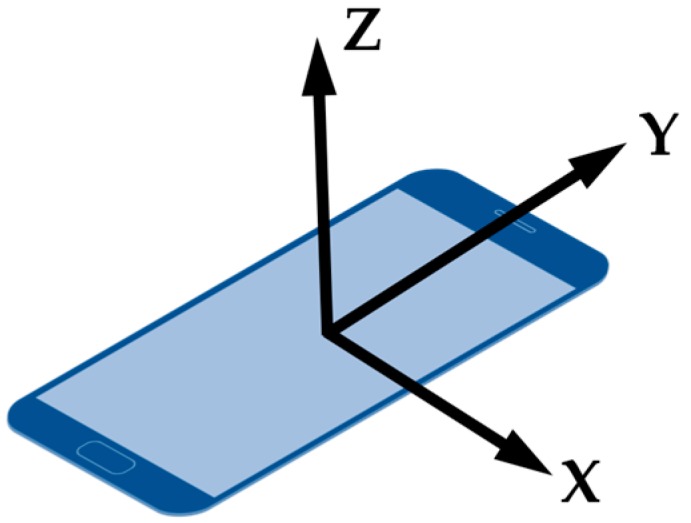
Three axes of magnetometer on smartphone.

WiFi and MFS data are stored as vectors which are step-indexed. However, the sampling rate of WiFi is slower than other sensors. Thus, it is not necessary to wait for WiFi data at each step. In other words, if WiFi is not available, set it to null in this step. Since MFS data provided by a smartphone (Android) are raw data (in μT) for each of the three coordinate axes which are shown as Figure 3. The value of each axis is influenced by orientation of a smartphone. So we compute the sum of the three-dimensional raw measurements to represent the MFS.

(3) $$M=\sqrt{m_{x}{}^{2}+m_{y}{}^{2}+m_{z}{}^{2}}$$

To guarantee the quality of data, we hold the smartphone in a fixed orientation as far as possible, such as ‘in front of chest with 60°’.

To combine scene information with location appropriately, we introduce the definition of transition points (TPs). Most of the transition points are in the boundary of scenes. In the data collection process, transition points are stop stations for scene label change. In most cases, TPs are start points or end points of indoor scenes. As shown in Table 1, (*x*_0_, *y*_0_) is a start point of Scene S1, while (*x*_1_, *y*_1_) is a start point of Scene S_2_ as well as end point of Scene S_1_, etc. For other data in the table, where *k*_1_, *k*_2_, …, *k*_(n−1)_ are numbers of steps detected in a scene, while *r*_1_, *v*_rssi 1_ and *v*_mfs 1_ are headings, vector of RSSI and vector of MFS at every step, respectively. A scene label can be seen as an attribute for TPs and steps.

**Figure 4:**
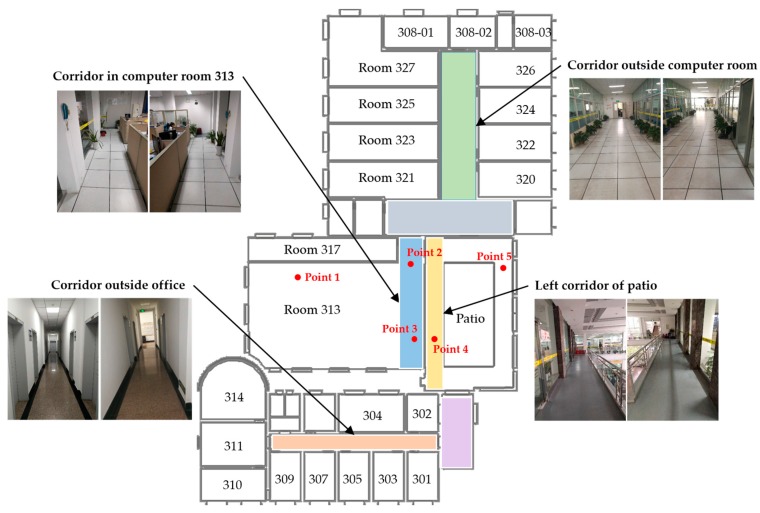
Examples of indoor scenes on the third floor of our laboratory. Two images are given in different walking directions.

Since deep learning is a kind of data-hungry method, a crowdsourcing method is designed to enlarge the training dataset. Images captured in the online phase also will be saved to server. Based on our recognition task, 20 different scene labels are defined which cover two floors in our laboratory. Examples of these scenes in the third floor of our lab are shown as Figure 4. The whole scene labels will be demonstrated in experiments. There are two guidelines for us to define indoor scenes.

- Firstly, locations with the same scene label must have same semantic significance. For instance, as shown in Figure 4, point 3 belongs to room 313 while point 4 belongs to patio, it is obvious they are in different scenes.
- Secondly, locations with the same semantic significance but in sensitive areas are preferrentially separated into different scenes. The sensitive areas here are more likely to achieve high error rate in the positioning phase. For instance, magnetic fingerprinting methods are difficult to distinguish locations at each side of a glass wall or door. As illustrated in Figure 4, the wall between room 313 and patio is a glass one. So the corridor inside room 313 and the corridor on the left of patio are defined as two different scenes to distinguish locations such as point 3 and point 4 even better.

#### 3.2.2. Fingerprints Processing

**Figure 5:**
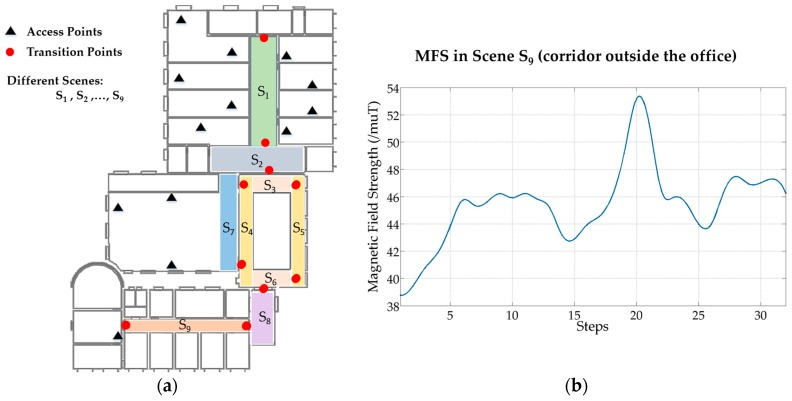
Environment information on the third floor of laboratory. (**a**) There are 13 access points, ten transition points and nine different scenes on this floor; (**b**) Change of MFS when walking in scene S_9_.

The final database of fingerprints contains two parts, one is TP-indexed, and the other is step-indexed. As described in the previous section, when walking around in a scene, WiFi RSSI and MFS are collected and organized by step. In order to improve the localization accuracy at start points, a user should stop at TPs to collect WiFi signal for at least 30 samples. Therefore, TPs can be seen as robust reference points and used as initial points for pedestrian dead-reckoning (PDR). As illustrated in Figure 5a, we defined nine scenes in the third floor of a laboratory, and the corresponding number of transition points is ten. Thus, we have ten stable reference points in this floor, and other locations are indexed by detected steps. In Figure 5b, change of MFS in a corridor (S_9_) is illustrated. It has shown that magnetic signal pattern is distinguishable in a five-step range, and it will be used to compute DTW distance in localization process.

### 3.3. Indoor Scene Model Training

In this section, fundamentals of a deep convolutional neural network is described, as well as a fine-tuning method for the indoor scene model with our own dataset. The training is implemented on the server side with GPU. Although methods for training a deep model on mobile devices have been proposed by some researchers [32,33], the capability of a modern smartphone is still not sufficient for this large computation requirement. In this paper, we use Caffe [34], an open source framework of deep learning, to implement model training and scene recognition. This framework is widely used in the computer vision community, and lots of trained Caffe models for different tasks with all kinds of architectures and datasets are provided in Caffe Model Zoo [35].

#### 3.3.1. Deep Convolutional Neural Networks (CNNs)

**Figure 6:**
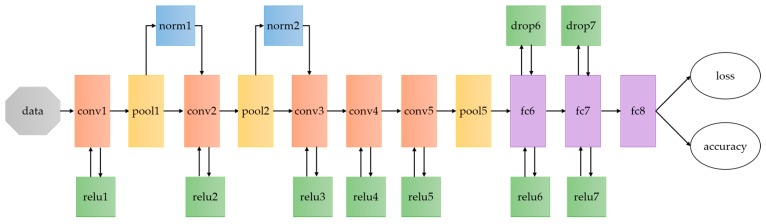
Architecture of CaffeNet. CaffeNet is a deep convolutional neural network modified from AlexNet with two differences: (1) training without the relighting data-augmentation; (2) pooling is switched to be done in front of normalization. CaffeNet is the base network for the proposed indoor scene model training.

As illustrated in Figure 6, the architecture of CNNs used in the proposed scheme is similar to CaffeNet [36], which is modified from AlexNet [27] and provided by Berkeley AI Research (BAIR). As we can see, this network consist of five convolutional layers (conv1, conv2, conv3, conv4, conv5) and three fully connected layers (fc6, fc7, fc8), which conv1, conv2 and conv5 are followed with pooling layers (pool1, pool2, pool5) as well as normalization (norm1, norm2, norm5). Note that the outputs of fc8 are 1000, which indicates the number of classes that need to be predicted for ImageNet datasets. For our task, there are 20 scenes in our dataset. Therefore, the output of fc8 is 20 in our networks.

For more details, specification of CaffeNet is given as Table 2. A convolution computation [37] can be denoted as Equation (4), where I is a two dimensional image as input, and K is a two-dimensional kernel.

(4) $$S\left(i,j\right)=\left(I,K\right)\left(i,j\right)=\underset{m}{\sum }\underset{n}{\sum }I\left(i+m,j+n\right)K\left(m,n\right)$$

(5) $$g\left(G,V,s\right)=\frac{\partial }{\partial K_{i,jk,l}}J\left(V,K\right)=\underset{m,n}{\sum }G_{i,m,n}V_{j,\left(m-1\right)\cdot s+k,\left(n-1\right)\cdot s+l}$$

In the training process, derivations with respect to the weights in kernel need to be computed. To do so, we can use a function as (5), where *s* stands for stride in convolution, *V* is multichannel image, *G* is a tensor which computed by cost function *J*. The last layer of CaffeNet is a loss layer, which utilize SoftmaxWithLoss denoted as (6), which $z_{j}$ stands for *j*th category in a recognition task.

(6) $$\tilde{l}=-log\left(\frac{e^{z_{y}}}{\sum_{j=1}^{m}e^{z_{j}}}\right)=\log\left(\overset{m}{\underset{j=1}{\sum }}e^{z_{j}}\right)-z_{y}$$

#### 3.3.2. Fine-Tuned Deep CNNs for Indoor Scenes Recognition

**Figure 7:**
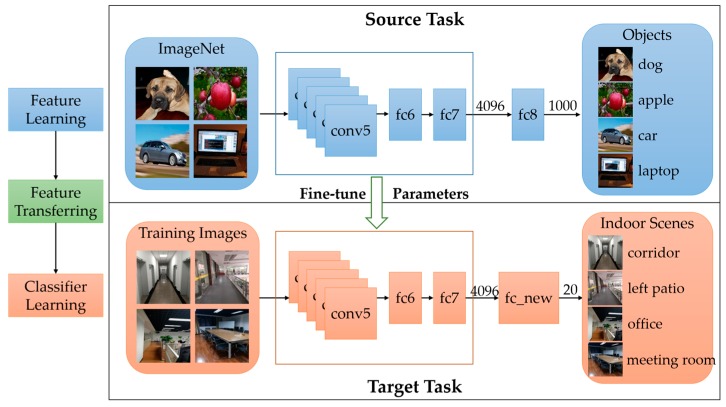
Fine-tuning a pre-trained network for indoor scene recognition. The outputs of final fully-connected layer for indoor scene task are 20 instead of 1000 in CaffeNet.

As mentioned before, deep learning needs large amounts of training data, which is difficult for a specific vision task like ours. However, for the representation power of CNNs, trained model based on ImageNet, which is a large-scale object dataset, can be used as initial value in our training process. Since high level features are already learned in hidden layers, it is not necessary to train our model from scratch. The fine-tuning process based on our dataset is shown in Figure 7. Features learned from ImageNet are used to be transferred to our classification task.

As demonstrated in Figure 7, outputs of the final layer are redefined to fit the categories of indoor scenes in our task. After getting the pre-trained network on ImageNet, we fine-tune parameters in the internal layers (conv1–fc7) to decrease the loss computed by the last layer (fc_new). Fine-tuning is a back propagation process which updates weights of parameters in hidden layers to decrease the loss. The most important tip for fine-tuning is the initial learning rate which need to be small to avoid over-fitting.

### 3.4. Location Estimation

In the online phase of the proposed system, locations can be estimated by three steps: current scene recognition, localization space narrowing and final localization estimation. 

#### 3.4.1. Indoor Scene Recognition

After training the indoor scene classifier, it will be deployed on a web server which can communicate with smartphones and receive recognition requests from clients. In our case, images from smartphones are captured automatically without special requirement for users, such as stop walking and deliberate focus on the current scene.

Before recognizing, preprocessing is needed to make test images meet the requirements of CaffeNet. The default configuration of CaffeNet is like this: format of image is BGR, pixel values start in the range of [0, 255] and subtract the mean pixel values of training data from them. The results present as top-5 probabilities with scene names, and we take top-1 as a result of scene recognition. However, it is a big challenge to allow image capturing to run all the time for it is extremely power-consuming. Thus, detection of proper time for capturing is needed. To solve this problem, we utilize TPs to detect potential scene change (Algorithm 1). k-Nearest Neighbor (kNN) algorithm is adopted during this process. Note that, the result of scene change detection is not the only activator for image capturing. It can also be activated by user input when necessary.

**Algorithm 1.** Ttransition Point Detection.Input: Samples of WiFi RSSIOutput: **TP’s postion**Begin: minimum distance equals infinite, nearest TP equals TP_1_1: **for** every TP fingerprint in database **do**2:  **while**
*n* < number of APs scanned **do**3:   compare mac address of every AP with TP fingerprint4:   **if** mac address is matched5:    number of matched APs ++6:   **end if**7:  **end while**8:  **if** number of matched APs > 39:   compute signal distance between samples and TP10:   **if** distance < minimum distance11:    update the minimum distance12:    update nearest TP13:   **end if**14:  **end if**15: **end for**16: compare scene label of nearest TP to current scene17: **if** not matched18:  **return ture**19: **else return flase**20: **end if**

#### 3.4.2. Space Narrowing and Localization

In this part, a fusion-based method is adopted to estimate user’s location. As described previously, all of the TPs or steps in a fingerprint database that have an attribute of an indoor scene. After the current scene is recognized, we use this attribute to reduce searching space of locations. In other words, users can be located in a sub-fingerprint database. Thus, localization error will decrease in sensitive areas which are hard to distinguish in other indoor localization systems. 

**Figure 8:**
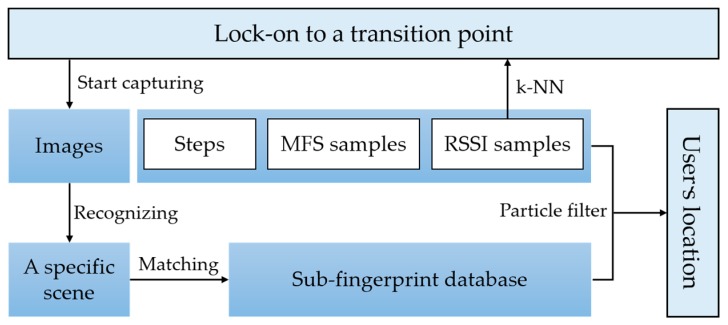
Procedures of localization estimation. Lock-on to a transition point is needed when the localization start. The detection of transition point utilize a k-NN algorithm.

The process of localization is shown in Figure 8. When the app starts, it will lock-on to a transition point and start recognition. After the current scene is detected, the fingerprint database is narrowed to this scene. Then, particle filter is used in this sub-fingerprints database. The transition point locked-on is regarded as a start point to spread particles. When a step is detected, the position of each particle is updated by corresponding step length and heading. And each particle is weighted by WiFi signal distance and magnetic dynamic time warping (DTW) distance. The final result is computed by the centroid of weighted particles. In this work, results of particle filter are constrained in a recognized scene. When the scene is changed, it will be locked-on to a new transition point.

For magnetic signal, two different places may have the same value since MFS is scalar. As can be seen in Figure 5b, MFS sequences are stable and distinguishable. Hence, MFS sequences are taken into account to measure similarities between samples and magnetic fingerprints. We use DTW to compute the similarity of two MFS sequences. DTW is a widely used algorithm in speech recognition which measures similarity between two different waveforms regardless of different speech speeds. Hence, different walking speed of users can be ignored by using the DTW algorithm in this case. However, it is better that length of MFS sequence is not too long or too short considering computation cost and accuracy. In the proposed system, a five-steps length is appropriate for localization considering balance of computation and accuracy. Figure 5b also has shown that a five-step length has good identifiability.

For WiFi signal, we compute the distance between scanned measurements and fingerprints by Equation (7).

(7) $$D_{wifi}=\frac{1}{n}\overset{n}{\underset{i=1}{\sum }}\left|rssi_{i}^{s}-rssi_{i}^{f}\right|$$

where $D_{wifi}$ denotes distance of two RSSI vectors, $rssi_{i}^{s}$ and $rssi_{i}^{f}$ are RSSI of *i*th AP from scanned measurements and database, respectively.

Then, fusion weight of each particle can be updated by Equation (8), where $k_{1}$ and $k_{2}$ denote tunable parameters, $D_{mag}^{i}$ and $D_{wifi}^{i}$ represent DTW distance and WiFi signal distance respectively.

(8) $$weight^{i}=e^{\frac{D_{mag}^{i}}{k_{1}}}\cdot e^{\frac{D_{wifi}^{i}}{k_{2}}}$$

Due to the noise of the WiFi signal, we set magnetic signal with higher weight than WiFi. In some detected steps, WiFi signal may be absent for the reason that the WiFi sampling rate is slower than other sensors. In that case, we only assign weight via magnetic signals.

## 4. Performance Evaluation

In this section, experiments of key modules in the proposed system are conducted. We test the accuracy of localization in a complicated indoor environment which contains glass walls and a rectangle patio between the second and third floors. Comparisons with two state-of-art schemes are also conducted. One is based on trajectories which combine PDR, WiFi and Magnetic. The other is IndoorAtlas [29], which is a commercial indoor localization platform based on a hybrid method, which combines PDR, WiFi, Magnetic, iBeacon and waypoints.

### 4.1. Experiment Setup

**Figure 9:**
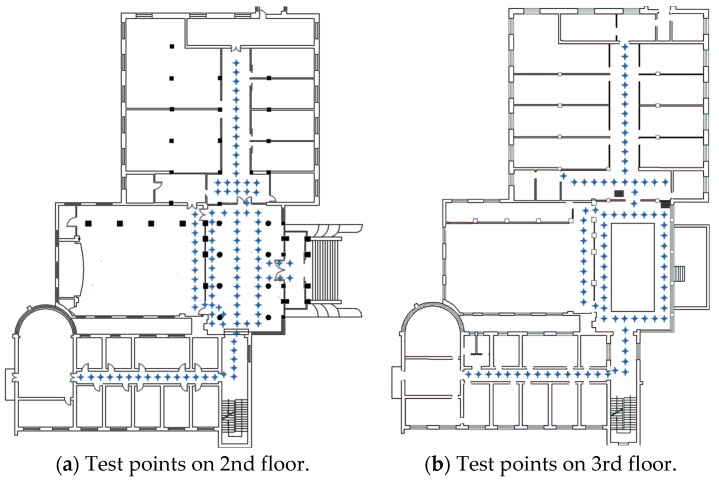
Test environment of the proposed system, which includes second and third floor of a laboratory in Wuhan University. (**a**) Floorplan of second floor, including 109 test points. (**b**) Floorplan of third floor, including 93 test points.

**Figure 10:**
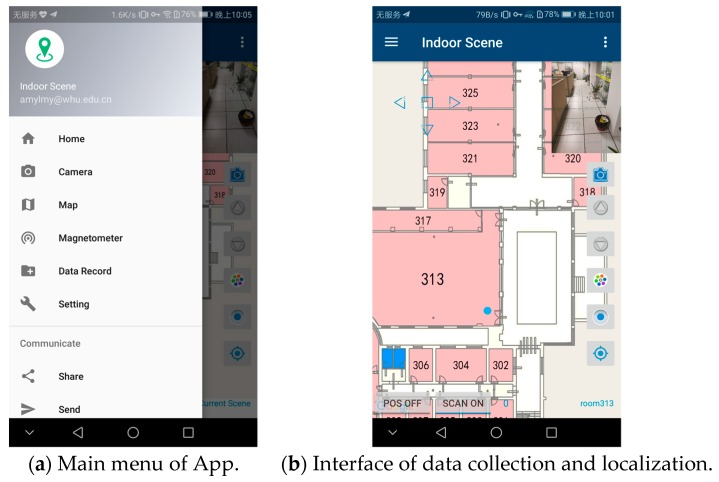
Interface of the proposed system on an Android phone. Before data recording start, we need to switch off the positioning mode, choose a current scene label and pick a start point on the map. Then, after switching on the scan mode, data recording will be activated.

The experiments were conducted on the second and third floors of a laboratory building, with a 57.5 m × 41.5 m floor area. In order to verify the effectiveness of the proposed system, areas that may be easy to be confused in other indoor localization system were taken into account in our experiments, such as hall, patio area and corridors on either side of a glass wall, etc. We test 202 points covering the main paths which users visit often. As shown in Figure 9, 109 test points belong to the second floor and 93 test points belong to the third floor. The ground truth is measured by a laser distance meter with a 1.5 mm precision. To evaluate the performance of the proposed system, data collection and localization modules were implemented on an Android platform (version 6.0) and tested with Huawei Honor 8. The interface of application is shown in Figure 10. 

**Figure 11:**
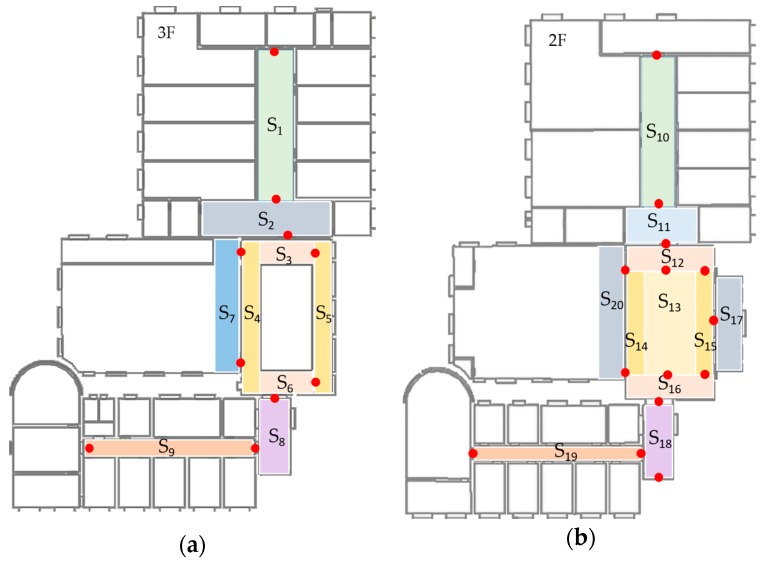
Definition of scene labels and transition points. (**a**) There are ten TPs and nine scenes in third floor; (**b**) 14 TPs and 11 scenes in second floor respectively.

The procedure of data collection has been mentioned in Section 3.2. In this experiment, only RSSIs stronger than −96 dBm are considered. Image data are collected in daytime and during a normal walking speed. The resolution of images are set to 240 × 320 which balances the quality and processing overheads. In this experiment, the number of indoor scenes are 20 and corresponding transition points are 24. Figure 11 shows the distribution of these scenes and transition points. The rule for us to divide these scenes is based on their semantic meanings and whether they are sensitive areas for localization. An example of different semantic meanings is that S_7_ is a corridor in front of a computer room and S_4_ is a corridor in a patio’s left side. An example of sensitive areas is that S_3_, S_4_, S_5_ and S_6_ are all around the patio, however, position errors occurred frequently in these areas due to their low signal discrimination. Without scene constraints, positioning results may bounce between these scenes. This phenomenon is particularly evident with IndoorAtlas and other signal-based methods. Examples of image details in these scenes can be found in Figure 4. For indoor scene recognition model, it was trained on a PC with an NVIDIA Titan X Graphic Card to get a fast training speed. To be noted that, other graphic cards or only with CPU are also supported. The architecture of the network is given in Section 3.3.

### 4.2. Performance of Indoor Scene Recognition

Training a deep learning model from scratch is time-consuming and the volume of data is hard to be satisfied. Nevertheless, we can still use our own dataset, a relatively small one, to train an effective model for our task. In this experiment, a trained model published by other researchers in Caffe Model Zoo [35] was utilized as initialization in the training process. The specification of this network is provided in Section 3.3.1. Weights of parameters in our model were adapted to fit the training data by back-propagation computing.

**Figure 12:**
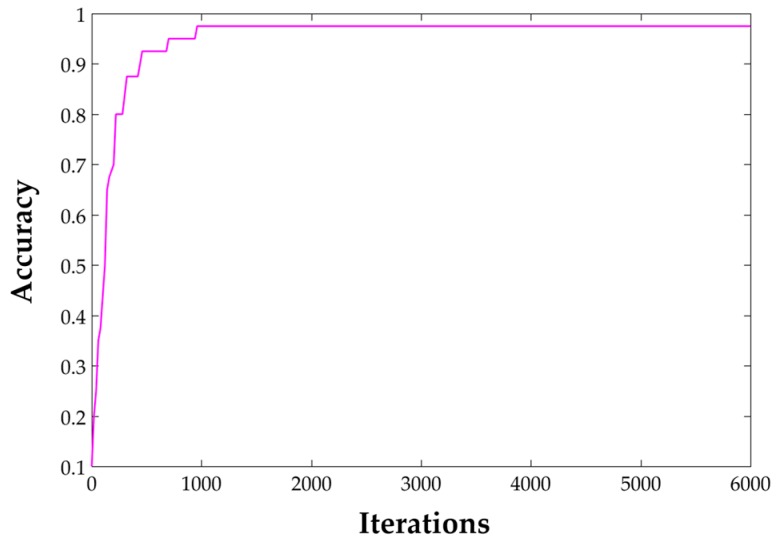
Accuracy change in training process.

**Figure 13:**
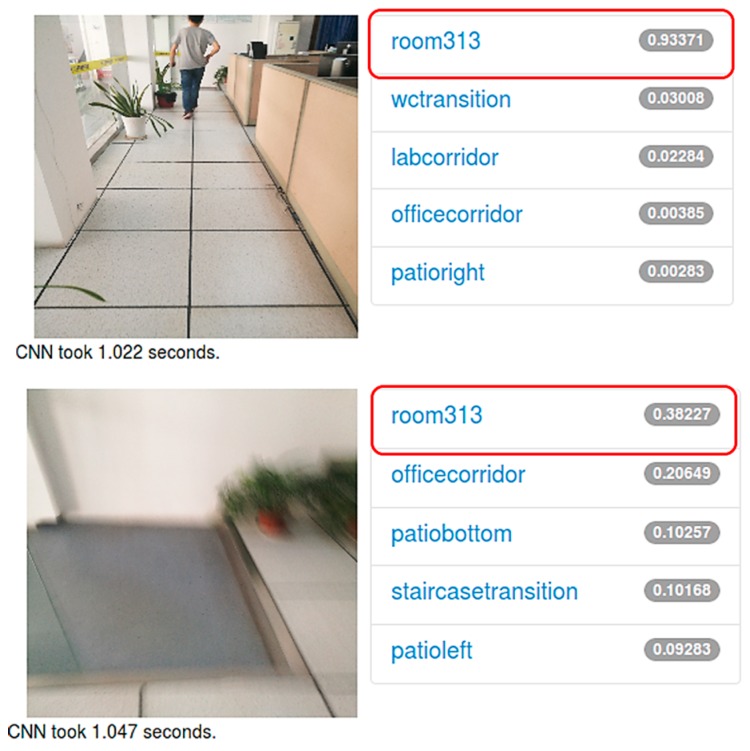
An example of indoor scene recognition with a bad-quality image. Both images were taken in room 313 of our lab. The second one is taken while a walking. The correct probabilities of these images are around 93.37% and 38.23% respectively.

In the training process, we gained a 97.5% accuracy after 6000 iterations on our own dataset, which includes 4800 images in total. Among them, 200 are used for training and 40 are used for validation in each scene. The changing trends of accuracy while training is displayed in Figure 12. As it can be seen, the speed of convergence is extremely fast. Testing of the trained model was also conducted in a test dataset. The report accuracy is 89.8% as shown in Table 3. It is important to be noted that in order to verify the generalization ability of this model, some bad-quality images are used in the test process. Figure 13 shows a recognition result on a web server. Both pictures were taken with a smartphone in room 313 of our lab. The first one was well-taken while the second one was heavily blurred for it was taken while walking. Even so, the top-1 result is correct and only it will be considered.

### 4.3. Localization Results and Analysis

In this part, comparisons of localization results will be demonstrated. We compared our results with two different schemes: (1) a trajectory-based method which involves fusion with PDR, WiFi and magnetic; (2) IndoorAtlas which is a commercial hybrid scheme with PDR, WiFi, magnetic, iBeacon and waypoints. 

**Figure 14:**
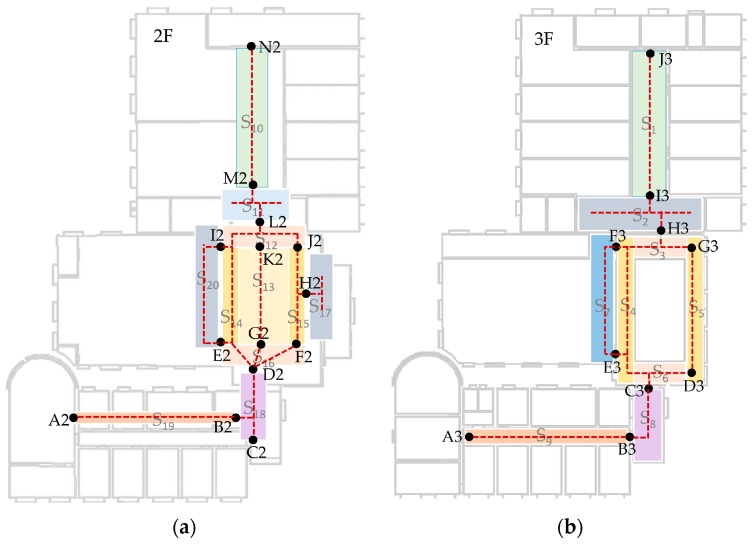
Paths to collect data for localization. (**a**) A2–N2 are TPs on the second floor and (**b**) A3–J3 are TPs on the third floor. In test phase, every path segments between two transition points were repeated at least three times.

Before localization, a fingerprint database needs to be built. In the proposed system, we recorded data by scenes while walking normally. The start point of a walking path needs to be a transition point (TP). As depicted in Figure 14, the path segments between different TPs (such as A2–B2, B2–C2, B2–D2 and so on) are recorded with scene labels (S_19_ and S_18_, etc.). The fingerprints at TPs are recorded by standing still and collecting at least 30 samples. The data format has been discussed in previous sections. For every path segment, step vectors are utilized to organize the fingerprint data. Every step must have a MFS (magnetic field strength) vector and may have a RSSI vector for the sample rate of WiFi is slower than the magnetometer. Headings also are stored in step vectors. Step length in this experiment is 0.66 m which is determined by an offline training.

As mentioned previously, after a scene image is captured by a smartphone, it will be sent to a server for recognizing and the result will be sent back to the smartphone immediately. In our fingerprint database, trajectory segments in a scene can be considered as a sub-fingerprint database which could be indexed by a scene label. After a scene is recognized, the system will estimate locations with corresponding sub-fingerprint database. The start point is determined by transition points. In fact, there are two scenarios in which the detection of transition point needs to be executed. The first one is when the app starts, we need to lock-on the user to a transition point as a starting point. The other is when the scene is changed, it is also necessary to determine the transition point to reset the start point for PDR to avoid accumulative error.

**Figure 15:**
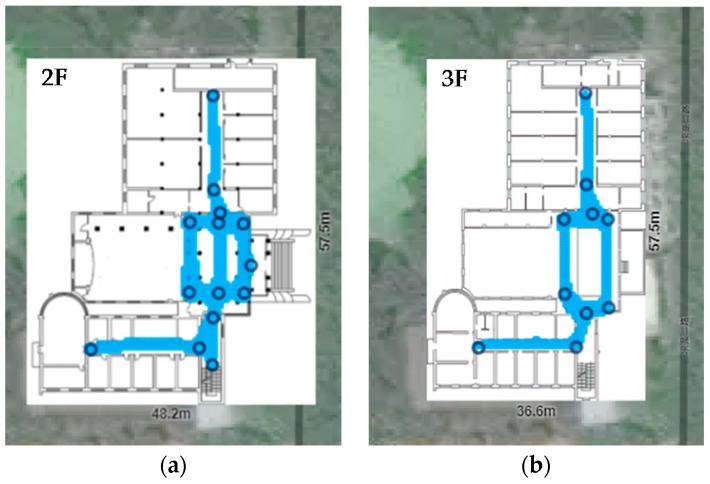
Coverage of site survey on IndoorAtlas. (**a**,**b**) are survey maps on the second floor and third floors, respectively. The black circles are waypoints we added in this experiment. A waypoint is pinpointed places on a map, and need to be check-in to define the actual position in data recording process.

In order to evaluate the performance of the proposed system, experiments of positioning without scene information were conducted. The first comparison is trajectory-based and involves fusion with multiple sensors such as IMU, WiFi and magnetometer. For comparison purposes, the storage format and collecting method of trajectories are similar to the proposed system. However, it does not contain any scene information. The second comparison is IndoorAtlas. The surveying method of IndoorAtlas is also by recording data while walking. As illustrated in Figure 15, mapping tool of IndoorAtlas requires users to define some waypoints for correction in the surveying phase. When a user arrives at a waypoint, a check-in operation needs to be carried out to tell the actual position of the user. The coverage of mapping is demonstrated with blue color in Figure 15. It covers all test points in this comparison experiment.

**Figure 16:**
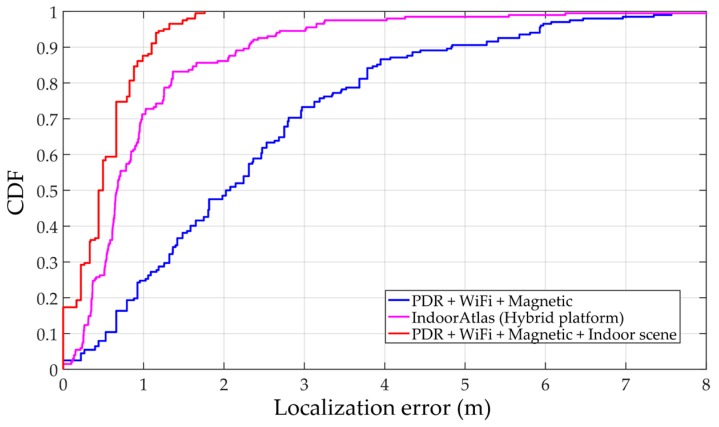
Empirical cumulative distribution function (CDF) of three different solutions. The proposed solution outperformed the conventional approaches without scene constraint.

Table 4 summarizes the error statistics of these three systems. The mean error of our system is 0.53 m which is better than the trajectory-based method without indoor scene and IndoorAtlas. The 95% accuracy in our system is 1.32 m which also has a significant advantage compared to others. The empirical cumulative distribution functions (CDFs) of these three systems are illustrated as Figure 16. With the constraint of indoor scene, the proposed system decreases the accumulative error of PDR, which is the main error source in trajectory-based systems. Moreover, it can also distinguish areas which have similar signal patterns, such as near a glass wall, patio etc. This is the reason why the proposed system can achieve higher accuracy than IndoorAtlas. Compared to our system, IndoorAtlas also defined waypoints to decrease the error of PDR which is similar to transition points in the proposed system. However, it fails to distinguish most of the positions in scenes S_7_ and S_4_ (Figure 11), since there is a glass wall between these two scenes. However, with the indoor scene recognition model, positions in these two scenes can be easily distinguished since sub-fingerprint databases corresponding to different scenes that are built in this paper.

## 5. Discussion

In this paper, we presented a multi-sensor fusion approach for indoor localization with scene constraint. A deep-learning method was adopted to recognize the current scene of the user. A recognition accuracy of 89.8% was achieved with our solution. A particle filter algorithm was then developed with constraints of scenes. Experimental results demonstrated that the positioning accuracy of our solution was 1.32 m at 95%, while that for the commercial product IndoorAtlas was 3.01 m in the same experimental environments. It is obvious that our solution outperformed conventional solutions without scene constraints. It is important to note that scene recognition cannot work well in dark environments or other bad visual conditions. Our system allows the user to switch the scene recognition module on or off to make sure it can be used in different kinds of environments.

Our solution has great potential to extend to a crowdsourcing-based method considering the data acquisition scheme. The trajectories from different users can be used to update and enlarge our fingerprint database, and images captured in the online phase will also be saved in our server for future model training since the volume of training dataset is important for deep neural networks. In the future, the problems that we need to solve for crowdsourcing trajectories are device heterogeneity and data integration. For indoor scene recognition models, a more efficient network architecture and more complicated environments need to be considered in future work.

## 6. Conclusions

In this paper, a scene-constrained indoor localization scheme has been proposed. The results and comparisons have shown that this scheme has a competitive performance compared to most current systems. We solved indoor localization problems especially in complicated indoor environments. Most systems fail to give a high positioning accuracy with easy deployment. In our system, we do not need to extract features in images compared to other vision-based methods. Deep learning methods only need to assign labeled training data to a pre-defined network; it will learn features automatically.

We are confident that the multi-sensor-fusion-based method may become the “killer” scheme for indoor localization systems without additional infrastructures. Based on the-state-of-art study of indoor localization, to the best of our knowledge, this work is the first to adopt deep learning to extract high level semantic information for indoor localization. This method is similar to human brain’s cognitive mode and has great potential in future research.

## Figures and Tables

**Table 1:** Information of data storage.

Scene Labels	TPs ^1^	Steps	Heading	WiFi RSSI	MFS ^1^
S_1_	(*x*_0_, *y*_0_)	*k*_1_	(*r*_1_, *r*_2_, ..., *r*_k1_)	(*v*_rssi 1,_ *v*_rssi 2,_ ..., *v*_rssi k1_)	(*v*_mfs 1,_ *v*_mfs 2,_ ..., *v*_mfs k1_)
S_2_	(*x*_1_, *y*_1_)	*k*_2_	(*r*_1_, *r*_2_, ..., *r*_k2_)	(*v*_rssi 1,_ *v*_rssi 2,_ ..., *v*_rssi k2_)	(*v*_mfs 1,_ *v*_mfs 2,_ ..., *v*_mfs k2_)
…	…	…	…	…	…
S_n−1_	(*x*_n_, *y*_n_)	*k*_n−1_	(*r*_1_, *r*_2_, ..., *r*_kn−1_)	(*v*_rssi 1,_ *v*_rssi 2,_ ..., *v*_rssi kn−1_)	(*v*_mfs 1,_ *v*_mfs 2,_ ..., *v*_mfs kn−1_)

^1^ TPs and MFS indicate transition points and magnetic field strength.

**Table 2:** Specification of CaffeNet.

Layers	Kernel Size	Stride	Pad	Output
conv1	11	4	0	96
pool1	3	3	0	96
con2	5	1	2	256
pool2	3	2	0	256
conv3	3	1	1	384
conv4	3	1	1	384
conv5	3	1	1	256
pool5	3	2	0	256
fc6	/	/	/	4096
fc7	/	/	/	4096
fc8	/	/	/	20

**Table 3:** Score of model for indoor scene recognition.

Model	Iterations	Time Cost	Top-1 (val)	Top-1 (Test)
CaffeNet	6000	23′39″	97.5%	89.8%

**Table 4:** Comparison of error statistics.

Method	Mean Error	Variance	95% Accuracy
PDR + WiFi + Magnetic ^1^	2.36 m	2.84	5.93 m
IndoorAtlas (Hybird platform)	1.08 m	2.69	3.01 m
PDR + WiFi + Magnetic + Indoor Scene ^2^	0.53 m	0.16	1.32 m

^1^ Trajectory-based method without indoor scene; ^2^ The proposed system.
